# Factors that influence the health of older widows and widowers—A systematic review of quantitative research

**DOI:** 10.1002/nop2.243

**Published:** 2019-02-26

**Authors:** Anne Lise Holm, Astrid Karin Berland, Elisabeth Severinsson

**Affiliations:** ^1^ Faculty of Health and Social Sciences Western Norway University of Applied Sciences Haugesund Norway; ^2^ Centre for Women’s, Family and Child Health, Faculty of Health and Social Sciences University of South‐Eastern Norway Kongsberg Norway

**Keywords:** bereavement, depression, emotional pain, health, older adults, systematic review, widowhood

## Abstract

**Aim:**

To examine factors that influence the health of older widows and widowers. The review question was: What is the evidence of the relationship between widowhood and health in older adults?

**Design:**

Systematic review.

**Data sources:**

Academic Search Elite, CINAHL, Medline (Ovid) and PubMed were searched for articles published between January 2013–December 2017.

**Review methods:**

A systematic review of quantitative research with a qualitative thematic analysis.

**Results:**

The selection process resulted in 12 studies. One of the themes that emerged was: emotional challenges related to experiences of bereavement, depression and anxiety, which was based on the sub‐theme social support as the main strategy for coping with emotional pain and suffering. The second theme was: struggling with poor physical health. The findings indicate that healthcare professionals need knowledge and skills to deal with the health consequences of widowhood in old age. Building community teams can prevent emotional and physical health problems, as well as reduce mortality.

## 1 INTRODUCTION

About 2.5 million people die in the United States each year (Centers for Disease Control & Prevention, 2014) where widowhood and bereavement (especially spousal loss) are disproportionately experienced by older adults (U.S. Federal Interagency Forun on Aging Related Statistics, 2012; Ghesquiere, Shear, & Duan, 2013). The death of a spouse has been described as having a negative impact on the health and well‐being of older community‐dwelling adults (Golden et al., 2009; Uhlenberg, 2009; Williams, Sawyer, & Allman, 2012). Marital status, quality and spousal age gap partly account for some of the health disparities related to widowhood (Choi & Vasunilashorn, 2014). Widowhood has been described as one of the most stressful events in life (Li, 2007). The literature also demonstrates that widowhood in old age is a dreaded phase of life due to its influence on health and well‐being (Agrawal & Arokiasamy, 2009; Aniruddha, 2013; Perkins et al., 2016).

Factors caused by social consequences that influence the health of older widows have mainly been described in Asian countries such as India, China and Japan, where older widows are exposed to neglect sexual abuse, violence and isolation (Agrawal & Arokiasamy, 2009). In these countries, older widows are considered to belong to their husband's family, whose members frequently view them as a burden (Aniruddha, 2013). Cheng, Chan, Li, and Leung (2014) found that having no children is significantly associated with depression among older widowed adults. Depression has been related to expectations and burden of support as well as being a care recipient (Tiedt, 2010, 2013; Tiedt, Saito, & Crimmins, 2016). Zhang and Li (2011) revealed the effect of marital status on depressive symptoms that was mediated by family support and moderated by the support of friends. Research from Mexico found that social integration can both mitigate and exacerbate depression among older widowed adults (Monserud & Wong, 2015). Thus, as mentioned by Aniruddha (2013), widowhood seems to have mental, social, behavioural and biological consequences, consistent with a stress‐inducing process. The meaning of bereavement, grief and mourning seems to differ across cultures, where most societies outline appropriate behaviours for those who are widowed based on socially constructed sets of norms (Robben, 2018). Cultural factors seem to influence health, and Lloyd‐Sherlock, Corso, and Minicuci (2015) found variations in the prevalence and timing of widowhood across countries such as China, Ghana, India, Russia and South Africa, in addition to associations between widowhood and being in the poorest wealth quintile of these countries. However, the evidence of the difference in impact across regions related to the cultural implications of widowhood on both individual and societal level is unclear. Widowhood is described as a cultural and gendered experience because the salience of different mechanisms linking widowhood to health may depend on local norms (Uhlenberg, 2009). Widowhood seems to trigger various health problems (Shear et al., 2011). The experience of losing a spouse appears to change over time (Williams et al., 2012).

Three systematic reviews (Holm & Severinsson, 2012; Lobb et al., 2010; Stahl & Schulz, 2014), one integrative review (Naef, Ward, Mahrer‐Imhof, & Grande, 2013) and two reviews (Merz & De Jong Gierveld, 2016; Nseir & Larkey, 2013), focused on different physical and/or mental health factors associated with bereavement in older widowed adults. Holm and Severinsson (2012) reviewed evidence about the emotional state of older widows. Lobb et al. (2010) aimed to clarify current knowledge to inform future planning and work in the area of complicated grief following bereavement. Stahl and Schulz (2014) examined the relationship between late‐life spousal bereavement and changes in routine health behaviours. Naef et al. (2013) determined key characteristics of the bereavement experience of older widowed persons. Nseir and Larkey (2013) examined the effect of interventions on the grieving process of older bereaved spouses. Merz and De Jong Gierveld (2016) investigated the role of family relationships through the lifespan in reducing loneliness among ever‐widowed older adults (i.e., persons who experienced the death of a spouse at some time during their life). These different reviews identified important factors that influence aspects of physical health (Stahl & Schulz, 2014); identity, mental health (Holm & Severinsson, 2012; Lobb et al., 2010; Naef et al., 2013; Nseir & Larkey, 2013); and family relationships (Merz & De Jong Gierveld, 2016). The reviews provided comprehensive evidence of factors that influence the health of older widows and widowers around the world. Such factors seem to have undergone a transformational change in recent years, especially in relation to the understanding of the human experience of loss as pointed out by Hall (2014). Older adults mostly describe the loss of a spouse as causing grief that decreases over time (Bonanno, Moskowitz, Papa, & Folkman, 2005). Despite our best efforts, we could find no review study examining factors that influence the health of older widows and widowers.

## 2 AIMS

The aim of this systematic review was to examine factors that influence the health of older widows and widowers. The review question was: What is the evidence of the relationship between widowhood and health in older adults?

## 3 METHODS

### 3.1 Design

A systematic review method was used to explore the available evidence (Moher, Liberati, Tetzlaff, Altman, & The PRISMA Group, 2009). Systematic reviews have a set of objectives that can be used to reproduce methodology including a systematic search to identify studies that meet the inclusion criteria. In addition, it is necessary to assess the validity of the findings and present a synthesis of the characteristics of the included studies (Moher et al., 2009).

### 3.2 Search strategy

**Figure 1:**
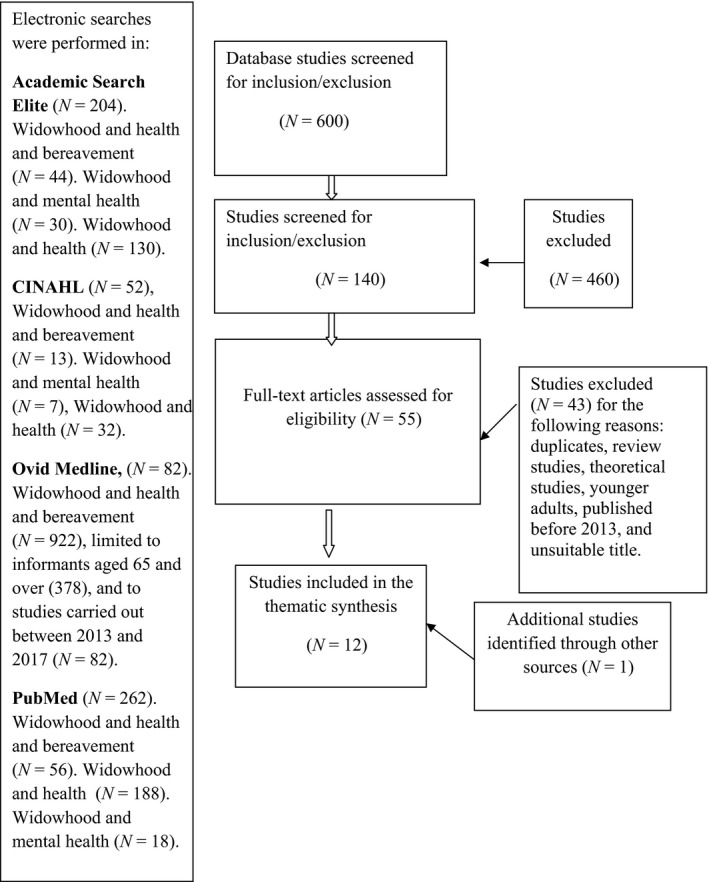
Flow chart of search outcomes and selection process of articles on the health status of widows and widowers (Moher et al., 2009)

Electronic searches were performed in Academic Search Premier (1), CINAHL (10), PubMed (191) and ProQuest (417) for the period January 2013–June 2017. The search words were as follows: widowhood, health, mental health and bereavement in various combinations. Boolean operators were used to maximize the searched terms for plurals, variations in databases and spelling (AND/OR/NOT) (Figure 1). A total of 600 abstracts were read and 55 studies (Figure 1) retrieved for further investigation. A manual search yielded one study.

The abstracts included studies that did not fulfil the inclusion criteria. The time period for this systematic review was limited to 5 years. The retrieval and selection process resulted in a total of 12 quantitative studies, presented in Figure 1.

### 3.3 Inclusion and exclusion criteria

Inclusion criteria were as follows: studies published between 2013–2017, older adults aged 60 years and over, and published in the English language in peer‐reviewed journals. Articles were selected based on the titles and keywords including concepts such as health, well‐being, mental health, depression and bereavement. As most relevant studies were quantitative, the authors decided to include only quantitative studies. Exclusion criteria were as follows: review studies, theoretical studies, younger adults and published before 2013.

### 3.4 Thematic analysis and synthesis

According to Holopainen, Hakulinen‐Viitanen, and Tossavainen (2008), the analysis of a systematic review can be either statistical or qualitative, depending on the material and purpose of the study. A thematic analysis was used to synthesize evidence related to the experience of bereavement and consequences for the health of widows and widowers (Bornbaum, Koras, Peirson, & Rosella, 2015; Guest, MacQueen, & Namey, 2012). Data were extracted and analysed by the first author (ALH), who read the extracts several times to identify relevant descriptions of the experience of bereavement and its consequences for the health of older widows and widowers. Themes were identified by inductive coding. The extracted data were synthesized by ALH. The draft synthesis was reviewed by the second (AKB) and third author (ES) and refined until agreement on the themes was achieved. Data that covered the same issue were coded and grouped together until convincing descriptions of the themes emerged. The process of determining suitable formulations was time‐consuming because the data were similar (Sandelowski, 2010).

## 4 RESULTS

### 4.1 Quality appraisal and methodological characteristics

Five studies were described as cross‐sectional and seven as cohort studies (Table 1). The studies were evaluated in accordance with the Critical Appraisal Skills Programme (CASP, 2014) (Table 2).

**Table 1:** Methodological characteristics of the included studies

	Researchers & Date/Country	Study design	Measurement	Confounding and control variables	Data analysis	Limitations
1.	Agrawal and Keshri (2014), INDIA	Longitudinal survey design based on data from 2004	Self‐report questionnaires: open‐ended questions. No descriptions of the validity or reliability of the instruments used	Age, residence, social group, religion, education, living arrangements; economic independence; monthly per capita expenditure percentile class	STATA 11.0	NM
2.	Burns et al. (2015), AUSTRALIA	Longitudinal survey design based on data from 1994 and follow‐up 16 years later	Subjective well‐being, Psychogeriatric Assessment Scales (PAS). Validation of the PAS depression scale where a score of 4 of more indicated depression in 80% of cases	Age; education (assessed by age on leaving school); when widowed; gender	STATA v.10	Future research needs to consider the range of social and individual factors that may moderate the capacity to be resilient to spousal loss. There is no indication of the extent to which the findings would be replicated in other Australian cities or in other national samples
3.	Carr et al. (2014), USA	Longitudinal prospective, design based on data from 1987–1988	Center for Epidemiologic Studies Depression (CES‐D) Scale, Symptom Checklist (SCL‐90R), Loss‐related anxiety, Despair, Bereavement Index. No description of the validity or reliability of the instruments used	Age, education, household income, self‐rated health, spouse's health, sex, depressive symptoms at baseline, life history of one major depressive episode	Bivariate analysis	Future studies should explore the direct effects of the wedding anniversary month on bereavement outcomes. The small sample size precluded an evaluation of two‐way interactions between date of interview and potential moderators including religious denomination, marital quality or cause of death
4.	DiGiacomo et al. (2013), AUSTRALIA	Longitudinal mixed method design. No information about the baseline interviews	Parkes’ Bereavement Risk Index‐Modified (BRI‐4), Short‐Form 12‐Item Health Survey (SF‐12v1), Depression, Anxiety and Stress Scales (Short Form) (DASS 21). The SF‐12 has undergone extensive validation and demonstrated good construct validity. The DASS has demonstrated reliability and the ability to document changes over time	Socio**‐**economic characteristics, living arrangements, years married, and number of children. Service utilization included the number of times a participant accessed different health professionals or services over the previous 6 months. Current diagnoses and medications	SPSS descriptive analysis	The small sample size precludes the opportunity of performing meaningful statistical tests and extrapolating to larger populations. Self‐report errors may have biased the findings, although repeated interviews provided opportunities to correct and contextualize reports. The mixed method longitudinal design facilitated collection of contemporaneous and contextual data useful for service planning
5.	Ghesquiere et al. (2013), USA	Longitudinal, prospective cohort design based on data from 1987–1988	Center for Epidemiologic Studies Depression (CES‐D) Scale. No descriptions of validity or reliability of the instruments used	Race, gender, education, income and single items on religious participation and importance of religious beliefs. Social network variables, positive emotional support from children, and instrumental support, anxious attachment style, an attachment anxiety composite.	STATA v.12	The small sample size could mean insufficient power to detect meaningful changes in some symptoms. The results may not be generalizable to other types of loss or to adults today. As the analyses lack both a control group and random or matched assignment to treatment conditions, causal inference is limited. Findings should be replicated
6.	Jeon et al. (2013), SOUTH KOREA	Longitudinal survey design. No information about the baseline interviews	Center for Epidemiologic Studies Depression (CES‐D10) Scale, Social tie measures, Korean Instrumental Activities of Daily Living Scale. No descriptions of validity or reliability of the instruments used	Age, education, income, experience of chronic diseases, disability, number of children and social participation	Multiple classification analysis	Small sub**‐**sample of widowers. Endogeneity remains the most serious threat to the validity of the findings with respect to social ties and depressive symptoms
7.	Panagiotopoulos et al. (2013), AUSTRALIA	Cross‐sectional design. No information about the year of sampling	Self‐report questionnaires, The Continuing Bonds Scale, Loneliness Scale (UCLA‐LS), Center for Epidemiologic Studies Depression (CES‐D) Scale. Religiosity and social support were moderate to high for both groups with reference to instrumental support from family. The reliability of the UCLA.LS Loneliness Scale (UCLA.LS) was high for both groups	Socio‐demographics: age, country of birth, length of time in Australia, preferred language, years widowed, years married, number of children, household composition, educational attainment, employment status, driving status, religious affiliation, self‐assessed financial stability	Independent sample *T* tests	The average duration of widowhood in the sample was 13 years. The mourning response to bereavement may have changed during this time (and hence impact on well‐being). Literacy issues among the Greek sample who had a lower educational level overall may have led to assistance from adult children when completing the questionnaire. This can be a source of bias and reporting errors. The quantitative approach meant that the participants were unable to differentiate between various forms of loneliness or understand the complexity and diversity of the widowhood experience. Future researchers must investigate the complex interplay between the socio‐historic, cultural, and individual life‐course and widowhood experience of ageing migrants in a foreign land
8.	Perkins et al. (2016), SOUTH KOREA	Cross‐sectional design based on data from 2011	Self‐report questionnaires, General Health Questionnaire. The General Health Questionnaire demonstrated validity and usefulness in several contexts	Marital status, age, caste and whether they lived with children in the same household, education, work status, household wealth quintiles	Gender‐stratified, multivariable, multilevel linear**, **and logistic regression analyses	Inability to examine objective markers of health and disease. The cross‐sectional nature of the data made it impossible to infer causality from the associational estimates. Future studies must clarify the relevance of marital status to health outcomes by collecting longitudinal data
9.	Spahni et al. (2015), SWITZERLAND	Cross‐sectional design based on data from 2012	Self‐report questionnaires; Center of Epidemiologic Studies Depression (CES‐D) Scale; Hopelessness Scale; De Jong Gierved Loneliness Scale; Satisfaction with Life Scale; Big Five Inventory (BFI‐10); the Resilience Scale. No descriptions of validity or reliability of the instruments used	Age, gender, level of education	Latent profile analysis (LPA)	Due to the cross‐sectional data, the opportunity that the identified classes reflect general instead of bereavement specific individual differences cannot be excluded. Larger samples necessary in future research
10.	Tiedt et al. (2016), USA	Longitudinal, design based on data from 1999, 2001, 2003, 2006 and 2009	Self‐report questionnaires; Center for Epidemiologic Studies Depression (CES‐D) Scale; Health. No descriptions of validity or reliability of the instruments used	Age, change in widowhood, social support, support availability**, **receipt of support	Baseline analysis	This study was limited by the fact that it did not use a weighted sample with equal periods of observations per individual. Controlling for multiple cross‐level interactions between the random sample slope for time and depressive symptoms predictors results in considerable multicollinearity
11.	Xu et al. (2017), USA	Cross‐sectional design based on data from 2006	Self‐report questionnaires; The Chinese version of the Geriatric Depression Scale Short Form had a reliability coefficient of 0.81. The control variables of the ADL and IADL instruments were 0.87 and 0.88, respectively	Age, gender, education, financial strain and functional health based on activities for daily living (ADLs) and instrumental activities of daily living (IADLs)	Descriptive and bivariate statistical analysis	Cross‐sectional data cannot detect the co‐occurrence or sequence between worry and depression, thus the associations found in this study need to be interpreted with caution. The measurements in future research must be more comprehensive
12	Zhou and Hearst (2016), CHINA, USA	Cross‐sectional design based on data from 2006	Self‐report questionnaires; Health‐related Quality of Life scale (QOL). QOL was described with good reliability and validity	Age, sex, highest level of education; whether suffering from chronic diseases and current marital status	Epidata 3.2a	The cross‐sectional design can detect associations but not cause and effect. The QOL has not been normalized for elderly Chinese populations, and responses may be biased for the 8.3% of elders for whom the questions were answered by caregivers

**Table 2:** Methodological quality assessment

Methodological quality assessment of the *prevalence *studies (CASP checklist)												
Questions in the checklist	1	2	3	4	5	6	7	8	9	10	11	12
Panagiotopoulos et al. (2013)	Y	Y	Y	Y	Y	NI	Y	NI	NI	–	U	Y
Perkins et al. (2016)	Y	U	U	U	Y	Y	Y	NI	NI	–	U	Y
Spahni et al. (2015)	Y	Y	U	U	Y	N	N	NI	NI	–	U	Y
Zhou and Hearst (2016)	Y	Y	U	U	Y	Y	Y	Y	N	–	U	Y
Xu et al. (2017)	Y	Y	Y	Y	Y	NI	Y	NI	NI	–	U	Y

Questions in the checklist	
1.	Was the aim/problem in the study clearly defined?
2.	Was the cross‐sectional design a suitable method to answer the aim and research question(s)?
3.	Was the population from which the sample was drawn clearly defined?
4.	Was the sampling method adequate?
5.	Was it explained whether (and how) the participants who agreed to participate are different from those who refused?
6.	Was the response rate adequate?
7.	Were the measurements shown to be valid and reliable?
8.	Were the procedures for data collection standardized?
9.	Was the statistical analysis appropriate?
10.	The conclusion of the studies (not included in this table)
11.	Can the results be transferred to practice?
12.	Do the results from this study support previous studies?

Methodological quality assessment of the *cohort *studies (CASP)												
Questions in the check list	1	2	3	4	5	6	7	8	9	10	11	12
Agrawal and Keshri (2014)	N	Y	U	U	Y	U	–	–	Y	Y	U	–
Burns et al. (2015)	N	U	U	U	U	U	–	–	Y	Y	U	–
Carr et al. (2014)	N	Y	U	U	Y	Y	–	–	Y	Y	Y	–
DiGiacomo et al. (2013)	U	Y	U	Y	Y	Y	–	–	Y	Y	Y	–
Ghesquiere et al. (2013)	N	Y	U	U	Y	Y	–	–	Y	Y	Y	–
Jeon et al. (2013)	U	Y	U	U	Y	Y	–	–	Y	Y	Y	–
Tiedt et al. (2016)	Y	Y	U	U	Y	Y	–	–	Y	Y	Y	–

Questions in the checklist	
1.	Did the study address a clearly focused issue?
2.	Was the cohort recruited in an acceptable way?
3.	Was the exposure accurately measured to minimize bias?
4.	Was the outcome accurately measured to minimize bias?
5.	Have the authors identified all important confounding factors in the design and/or analysis?
6.	Was the follow‐up of participants complete and long enough?
7.	What are the results of this study? (not included in this table)
8.	How precise are the results? (not included in this table)
9.	Do you believe the results?
10.	Can the results be applied to the local population?
11.	Do the results of this study fit with other available evidence?
12.	What are the implications of this study for practice? (not included in this table)

Note

N: no; NI: not identified; U: uncertain; Y: yes.

Methodological characteristics of the included studies are described in Table 1. The cross‐sectional studies comprised data from 2006–2012 except for one that lacked information about the year when the baseline interviews were conducted (Panagiotopoulos, Walker, & Luszcz, 2013). Old baseline data can be a limitation and lead to a risk of bias, thus threatening validity, reliability (Polit & Beck, 2012) and distorting the results. Such data could reflect general and not specific individual differences (Spahni, Morselli, Perrig‐Chiello, & Bennett, 2015). This could be related to the history threat described by Polit and Beck (2012) as a criterion of internal validity associated with the time factor, which means that the data may have changed since collection. The cross‐sectional studies used self‐report questionnaires (Table 1). However, these studies contain no discussion about how self‐report questionnaires might have increased response bias (Polit & Beck, 2012). Three studies provided no information about the response rate (Table 3), which can also increase the risk of bias. Two of the cross‐sectional studies have methodological recommendations about future research (Table 1). In two of the studies, the use of a longitudinal design in future research is recommended (Perkins et al., 2016; Spahni et al., 2015).

Four of the cohort or longitudinal studies were based on old data from 1987–2004 (Table 1). Table 2 reveals that six studies have no clear focus, which can increase exposure and outcome bias. According to Schneider, Whitehead, Elliott, Lobiondo‐Wood, and Haber (2007), a cohort study is generally prospective and employs an epidemiological approach in the direction of exposure to outcome, or cause to effect. Minimizing bias is important in all cohort studies. A problem in this systematic review was that most of these studies described their design differently and not very clearly (Table 2). In cohort or longitudinal studies, data are collected at two or more time points over an extended period (Polit & Beck, 2006). The long duration of data collection is a disadvantage due to the cost in terms of time, effort and resources. Another disadvantage is the threat to internal validity including “testing,” “mortality” (loss to follow‐up) and the influence of confounding variables (Schneider et al., 2007). Social desirability bias can be possible, where informants respond in a way they believe is congruent with the researchers’ expectations. Four studies described the small sample size (Tables 1 and 2), which can increase bias. Two studies lacked consideration of how to minimize bias (Agrawal & Keshri, 2014; Burns, Browning, & Kendig, 2015). Four studies were based on self‐report questionnaires (Table 1). According to Shadish, Cook, and Campbell (2002), self‐reports can increase the likelihood of response bias. Self‐reports can be seen as a retrospective data sampling method that increases bias due to the informants’ poor memory and inability to remember what actually happened (Polit & Beck, 2012), thus decreasing validity. Self‐reports can also be related to unmeasured confounders. Most of the studies lack references to methodology.

Two studies were described as prospective (Table 1). A prospective design begins with independent variables and looks forward to the effect (Polit & Beck, 2006).

Six of the cohort studies identified important confounding factors in the design and/or analysis (Tables 1 and 2). The follow**‐**up of participants was complete and of adequate duration (Table 2). No descriptions of validation or the reliability of the measurement instruments used were provided in five of the cohort studies (Table 1). Four of the studies did not refer to the response rate (Table 3). Generalization or external validity was not mentioned in five studies, which can constitute a limitation (Table 2).

**Table 3:** Main results of the studies included in the systematic review

	Researchers, year and country	Aim	Study sample	Main results	Key aspects that contribute to the themes	
					1) Emotional challenges related to experiences of bereavement, depression, and anxiety. Sub‐theme: social support as the main strategy for coping with emotional pain and suffering	2) Struggling with poor physical health
1	Agrawal and Keshri (2014), National Sample survey (NSS), INDIA	(a) compare the patterns of disease prevalence among older widows in terms of communicable, non‐communicable and other diseases, (b) treatment seeking behaviour of older widows, and (c) study the variations with reference to socio‐economic and demographic factors	Community‐dwelling adults, *N* = 10,111 widows, 60 years or older, RR:NM	Overall morbidity was 12% greater among older widows compared to older widowers. Likelihood of seeking healthcare services for reported morbidities was substantially lower among older widows		Multimorbidity or chronic diseases caused the participants to struggle with poor physical health and risk of mortality
2	Burns et al. (2015), Melbourne Longitudinal Studies on Healthy Ageing, (MELSHA), AUSTRALIA	Incorporating measures of subjective well‐being, defined in terms of the presence of positive affect, the absence of negative affect and clinically relevant depressive symptoms	Community‐dwelling older adults, *N* = 652, 65 years or older, either married or widowed, *N* = 75. *N* = 89 became widowed over the survey period, RR:70%	Widowhood was related to decline in positive affect. Otherwise, no long‐term impact of widowhood on negative affect or depressive symptomology was reported	The health of widows and widowers was described as poor and dominated by experiences of loss, grief and depression	
3	Carr et al. (2014), Changing Lives of Older Couples (CLOC) study, USA	To assess whether seasonal variation in psychological symptoms is more acute among the recently bereaved	Community‐dwelling older adults, 65 years or older. *N* = 297 (*N* = 210, *N* = 87 controls), *N* = 370 (*N* = l84, *N* = 186 controls). gender; NM, RR:NM	Widowed persons reported heightened psychological distress when interviewed during the month of their late spouse's birthday, a postholiday period (January), and in June, a month associated with wedding anniversaries and graduations in the United States. The distressing effects of special occasions on psychological symptoms were only evident within the first 6 months loss and were not apparent at the 18‐month follow‐up	January interviews revealed significantly higher levels of experiences of loss, anxiety and depression	
4	DiGiacomo et al. (2013), AUSTRALIA	To describe experiences of older recently widowed women by providing a snapshot of health and health service use during the early widowhood period	Community‐dwelling women *N* = 21, 65 years or older, RR:NM	The majority of participants scored within normal ranges for depression, anxiety and stress, yet a subset had elevated levels of each of these constructs (37%, 27% and 19%, respectively) throughout the study period. Positive self‐reports of general health predominated, despite the fact that 86% of participants were living with one or more chronic conditions and taking an average of four medications per day. 76% experienced exacerbations of existing conditions or were diagnosed with a new illness in the early bereavement period, leading to planned and unplanned hospitalization and other health service use		Multimorbidity or chronic diseases caused them to struggle with poor physical health
5	Ghesquiere et al. (2013). The CLOC study, USA	To examine whether utilization of family doctors was associated with reductions in grief, depression or anxiety among those with complicated grief and/or depression	Community‐dwelling, adults, *N* = 89, *N* = 24 widowers, *N* = 65 widows, 65 years or older, RR:NM	The analysis indicated that seeking support from a family doctor at wave 1 was not associated with changes in anxiety, depression or grief severity at wave 2 (*p *> 0.05). However, support group use was associated with reductions in grief severity (−8.46, *p *< 0.05), and religious leader support‐seeking was associated with reductions in depression severity (−10.12, *p *> 0.01). The findings imply that physician care for grief may not be effective, and support group referral may be helpful. Physicians may benefit from training in recognizing and providing appropriate referrals for bereavement‐related distress	35% of the widowed participants met the criteria for complicated grief and depression. Widows and widowers need social support	
6	Jeon et al. (2013). The Korean Longitudinal Study of Aging (KLoSA), SOUTH KOREA	To examine the impact of social ties on the relationship between widowhood and depressive symptoms among the older Korean population	Community‐dwelling adults, 60 years and older. *N* = 4,422 *N* = 1,952 widower**s**, *N* = 2,470 widows, RR: 75.4%	The quality of the relationship between women and their children accounted for 51.52% of the difference in depressive symptoms between married and widowed women, but only 11.36% between married and widowed men		Multimorbidity or chronic diseases caused them to struggle with poor physical health
7.	Panagiotopoulos et al. (2013), AUSTRALIA	To examine the well‐being of older migrant widows from two groups in South Australia: British‐born and Greek‐born Australian migrants, who had been widowed for an average of 13 years	Community‐dwelling migrant widows, *N* = 121, *N* = 61, British‐born, *N* = 60, Greek‐born, 60 years or older, RR:NM	Greek‐born widows exhibited higher levels of mourning rituals, continuing bonds and religiosity than the British‐born. Both groups perceived similarly high levels of familial social support. Greek‐born widows also reported worse self‐rated health and increased symptoms of depression and loneliness compared to the British‐born widows. The impact of widowhood on well‐being may be greater for non‐English‐speaking migrants who are ageing outside of their country of origin, and who, despite residing in an English‐speaking host country for several decades, have retained the linguistic, cultural and religious practices and traditions of their home country	The health of widows was described as poor, dominated by experiences of loss, grief and depression	Widows need support from their children
8	Perkins et al. (2016), SOUTH KOREA	To examine the relationship between widowhood and self‐rated health, psychological distress, cognitive ability, and four chronic diseases before and after adjusting for demographic characteristics, socio‐economic status, living with children, and rural‐urban location for men and women separately	Community‐dwelling adults, *N* = 9,615, *N* = 4,562 widowers, *N* = 5,053 widows. 60 years or older. RR:93%	Being widowed as opposed to married was associated with worse health outcomes for women after adjusting for other explanatory factors. Widowhood in general was not associated with any outcomes for men except for cognitive ability, although men who were widowed for 0–4 years were at greater risk of diabetes compared to married men. Moreover, recently widowed women and women who were widowed long‐term were more likely to experience psychological distress, worse self‐rated health and hypertension, even after adjusting for other explanatory variables, whereas women widowed for 5–9 years were not		Multimorbidity or chronic diseases caused them to struggle with poor physical health
9.	Spahni et al. (2015), SWITZERLAND	To identify patterns of psychological adaptation to spousal loss in old age and to shed light on the role of intra‐ and interpersonal resources and contextual factors as discriminant variables in these patterns	Community‐dwelling adults, *N* = 402, *N* = 174 widowers, *N* = 228 widows, *N* = 618 controls, *N* = 306 widowers, *N* = 312 widows, mean age 73.82 years, 60 years and older, mean age 74.41 years, RR:32%	The outcomes of depressive symptoms, hopelessness, loneliness, life satisfaction and subjective health revealed three different groups in the widowed sample: resilient (54%), copers (39%) and vulnerable (7%). The most important variables for group allocation were intrapersonal resources**,** psychological resilience and the Big Five personality traits, but also the quality of the former relationship and how the loss was experienced	Differences in psychological resilience between the three profiles were statistically significant. The resilient group and the copers group showed more positive emotional valence concerning experience of loss than the vulnerable group	
10.	Tiedt et al. (2016), The Nihon University Japanese Longitudinal Study of Aging, (NUJLSOA), USA	To examine the relationships among depressive symptoms, transitions to widowhood, worsening health and family support in Japan over 10 years	Community‐dwelling adults, *N* = 2,636, gender: NM, mean age 73 years, RR:74.6%	Becoming widowed correlated with increased depressive symptoms and this relationship was weaker among women than men. Continuous widowhood was associated with fewer depressive symptoms over time. Transition to co**‐**residence with sons and daughters was correlated with reduced depressive symptoms. Self‐reported health and difficulty with activities of daily living were predictors of depressive symptoms over time. The findings suggest the importance of new research on household transitions, availability and proximity of family caregivers, and social embeddedness as a protection against depressive symptoms	Widows and widowers need support from their children	Multimorbidity or chronic diseases caused them to struggle with poor physical health
11	Zhou and Hearst (2016), CHINA	How widowhood affects QOL of Chinese elders in rural areas. To explain subsequent morbidity and mortality and suggest ways to promote the health of these older people	Community‐dwelling adults, *N* = 1,060, *N* = 1,925 controls, *N* = 310 widowers, *N* = 750 widows, 60 years and over, RR:NM	The physical and mental health of elderly widows and widowers declined with age. Widowed men had lower physical component summary scores and mental component summary scores than married men. Widowed women had lower physical component summary scores, but the differences in mental health summary scores were not statistically significant. Widowhood was associated with lower scores overall. Support from children was associated with better QOL and, based on interaction analysis, appeared to mitigate the negative effects of widowhood		Multimorbidity or chronic diseases caused them to struggle with poor physical health
12	Xu et al. (2017), NSS of the aged Population in urban/rural China, USA	To examine whether worry about not having a caregiver in old age was associated with depressive symptoms among widowed Chinese older adults, including the moderating effects of self‐perceived family support	Community‐dwelling adults, *N* = 5,331, *N* = 1,641 widowers, *N* = 3,690 widows, 60 years or older RR:97.1%	Individuals who were worried about not having a caregiver reported significantly higher levels of depressive symptoms. Feeling that their children are filial, having instrumental support from children and having only daughters moderated the effects of worry about not having a caregiver on depressive symptoms	Widows and widowers need support from their children	

Note

*N*: number of participants; NA: not assessed; NM: not mentioned; QOL: quality of life; RR: response rate.

Demographic characteristics (Table 4). Six studies have samples from India, South Korea, China and Japan, while another six studies contain samples from Australia, the Unites States and Switzerland. Nine studies include information about the educational level of the sample. All studies contain information about the mental and physical health status of those in the sample, and six give information about their living conditions. Four studies contain no information about how long those in the sample have been widowed. Four studies include information about financial status.

**Table 4:** Demographic characteristics of the included studies

Researchers	Ethnicity	Education	Health status	Living situation	Widowed	Financial status
Agrawal and Keshri (2014), INDIA	Indian	Some of the sample had a high school education and all had completed middle school	Mental disorder, diarrhoea, fever of unknown origin, tuberculosis, whooping cough, kidney disease, skin diseases, gastritis, other communicable diseases, cataract, eye diseases, joint and bone disorders, asthma, diabetes mellitus, respiratory diseases including the ear, hypertension and other non‐communicable diseases	Living with children and other relatives	NM	Economically dependent older widows reported a greater prevalence of morbidities compared with economically independent older widows
Burns et al. (2015), AUSTRALIA	NM	A substantial number left school between the age of 13–14 years (47%), 15–16 (31%), with 19% leaving school after the age of 16 years	Depression, no measurements of physical health or diseases	NM	Widowed for 4 years prior to the baseline observation	NM
Carr et al. (2014), USA	NM	Educational attainment was 11.7 years	Depression, anxiety, despair, no measurements of physical health or diseases	NM	NM	NM
DiGiacomo et al. (2013), AUSTRALIA	NM	NM	Depression, anxiety, and stress. At least one chronic condition	The participants had an average of two children, five widows had no children	2–47 months since the loss in the 1st interview, 8–53 in the 2nd and 13–59 in the 3rd	NM
Ghesquiere et al. (2013), USA	White 84.7%	44.2% of the sample had no high school education, 28.4% had a high school education, 17.6% had some college education and 9.8% had completed their college education	Depression, grief, no measurements of physical health or diseases	NM	NM	NM
Jeon et al. (2013), SOUTH KOREA	Korean	79.7% of the sample had an elementary school education or less	Depression, hypertension, diabetes, cancer, lung disease, heart problems, stroke, arthritis, and gastrointestinal maladies in addition to difficulties in performing activities of daily living	NM	NM	Widows have significantly lower levels of economic and social activity. Widowers reported a higher average monthly income than widows
Panagiotopoulos et al. (2013), AUSTRALIA	White	The sample included individuals with higher educational attainment	Depression, loneliness. Well‐being (self‐rated health), no information on diseases	105 of the participants lived alone, 13 lived with their children, three with relatives, friends	British‐born widows had been widowed for 14 years. Greek‐born widows had been widowed for 12 years	NM
Perkins et al. (2016), SOUTH KOREA	Korean	31.3% of the sample had no education, 21.8% had <5 years, 32.5 had 6–10 years and 14.4% had over 11 years of education	Mental disorder, hypertension, diabetes, asthma, arthritis	71% of the widowers lived with their children. 72.6% of the widows lived with their children	4% of widowers had been widowed for 0–4 years, 4% for 5–9 years, 6% for 10 years of more. Among widows, 14% had been widowed for 0–4 years, 13% for 5–9 years and 34% for 10 years or more	NM
Spahni et al. (2015), SWITZERLAND	White	The participants had completed secondary (58%), tertiary (28%) or primary (14%) level education	Depression, hopelessness, loneliness. No measurements of physical health or diseases	NM	The sample had been widowed for a maximum of 5 years	NM
Tiedt et al. (2016), USA	Japanese	NM	Depression, heart diseases, cancers, cerebrovascular ailments, high blood pressure, respiratory illnesses, digestive illnesses, diabetes, renal/urinary tract ailments, ailments of the liver/gall bladder, arthritis, chronic back pain, fractures/fissures, other fractures, and osteoporosis. Difficulties in performing the activities of daily living	NM	32% of the sample was widowed at baseline	NM
Xu et al. (2017), USA	Chinese	41.1% of the sample had some form of education	Mental health, general health	62.1% of the participants lived with their children, 1.9% did not have any children, 13.2% only had sons, 7.1% only had daughters, and 77.8% had both sons and daughters	NM	60.9% of the participants were financially stable. 56.9% received financial support from their children
Zhou and Hearst (2016), CHINA	Chinese	NM	Depression, difficulties in performing the activities of daily living	Most of the participants lived with their children	5.7% had been widowed for less than a year and 71.9% had been widowed for over 5 years	40% of the participants relied on their children as the main source of financial support

Note

NM: not mentioned.

### 4.2 Characteristics of the substance of the quantitative studies

The key aspects that contributed to the evidence from the 12 quantitative studies are presented in Table 3. One of the themes that emerged was: emotional challenges related to experiences of bereavement, depression and anxiety based on the sub‐theme social support as the main strategy for coping with emotional pain and suffering. The second theme was: struggling with poor physical health.

#### 4.2.1 Emotional challenges related to experiences of bereavement, depression and anxiety

Eight of the included studies describe the health problems of widows and widowers as influenced by the experience of bereavement, depression and anxiety (Burns et al., 2015; Carr, Sonnega, Nesse, & House, 2014; DiGiacomo, Lewis, Nolan, Phillips, & Davidson (2013); Ghesquiere et al., 2013; Jeon, Jang, Kim, & Cho, 2013; Panagiotopoulos et al., 2013; Spahni et al., 2015; Xu, Li, Min, & Chi, 2017).

Four studies found that the participants suffered from depression, anxiety and/or complicated grief disorder (Burns et al., 2015; Carr et al., 2014; DiGiacomo et al.,^2013^; Ghesquiere et al., 2013; Panagiotopoulos et al., 2013). Those interviewed within a month of their late spouse's birthday reported despair and depression (Carr et al., 2014). In the survey by Ghesquiere et al. (2013), 35% of the widowed participants met the criteria for depression and/or complicated grief disorder on the first measurement occasion, when it was found that 77 met the early criteria for complicated grief disorder, 27 had co‐occurring depression, and 12 met the criteria for depression without complicated grief disorder. Of this group, 65 participants were interviewed later and 22 met the criteria for complicated grief disorder, of whom five had co‐occurring depression and nine depression without complicated grief disorder. The study by Panagiotopoulos et al. (2013) revealed that Greek‐born widows differed from their British‐born counterparts, scoring significantly lower on self‐rated health and significantly higher on depression and loneliness.

Spahni et al. (2015) examined the differences between a widowed and a married sample in relation to the experience of loss. Three groups were identified and labelled: the resilient group, the vulnerable group and the coper group. The resilient group (*N* = 215) showed a significantly higher level of extraversion, lower neuroticism and a higher level of consciousness, agreeableness and openness than the copers (*N* = 155) and the vulnerable group (*N* = 30). All differences in psychological resilience between the three profiles were statistically significant. In addition, the resilient group reported a significantly longer time since their loss than the copers. The resilient group and the coper group showed more positive emotional valence concerning the experience of loss than the vulnerable group (Spahni et al., 2015).

One study found that widows reported more depressive symptoms than widowers (Tiedt et al., 2016).

##### Social support as the main strategy for coping with emotional pain and suffering

Eight studies revealed that the older widows and widowers managed their emotional pain and suffering thanks to support described as social bonds, family support or support from friends and that their health problems made daily life a struggle (Agrawal & Keshri, 2014; DiGiacomo et al., 2013; Ghesquiere et al., 2013; Jeon et al., 2013; Perkins et al., 2016; Tiedt et al., 2016; Xu et al., 2017; Zhou & Hearst, 2016). In four studies, the participants needed support from their children and families (Jeon et al., 2013; Panagiotopoulos et al., 2013; Tiedt et al., 2016; Xu et al., 2017), as well as from other people (DiGiacomo et al.,^2013^; Ghesquiere et al., 2013). Three types of social tie were described, namely contact with statistically significant others, cohabitation with married children and relationships with their children (Jeon et al., 2013). Two studies reported more instrumental support and companionship from sons than from daughters and daughters‐in‐law (Tiedt et al., 2016; Xu et al., 2017). Transitions to widowhood exhibited a correlation with depressive symptoms, while receiving support was related to a lower level of depressive symptoms (Tiedt et al., 2016; Xu et al., 2017). Instrumental support from daughters correlated with increased depression, while companionship from a daughter**‐**in‐law correlated with reduced depressive symptoms (Tiedt et al., 2016). Widowed elders who were living with sons and daughters derived clear benefits compared with those not living with children (Tiedt et al., 2016). The perception of children's filial piety (caring ability) was significantly related to a lower level of depressive symptoms (Xu et al., 2017). Living with sons and daughters was found to reduce depressive symptoms. The results indicated that worry about not having a caregiver was significantly associated with depression. A study from Australia found that the participants perceived a high degree of emotional support from family and friends (Panagiotopoulos et al., 2013). The Greek‐born sample perceived significantly greater instrumental support from family, whilst the British‐born sample described greater support from friends. For the British‐born, loneliness was significantly correlated with family emotional and instrumental support, while emotional support from friends was significantly correlated with loneliness and depression. For the Greek‐born, well‐being was significantly correlated with family emotional support, with strong correlations between the variables of health, depression and loneliness compared with the British‐born sample. Loneliness was also significantly correlated with family instrumental support, while emotional support from friends was reported to a lesser degree compared with the British‐born sample. The value of being part of a support group was described in two studies (DiGiacomo et al., 2013; Ghesquiere et al., 2013). Being part of a support group was associated with reduced grief severity, while support from a religious leader was associated with reduced depression severity. However, support from a family doctor was not associated with changes in anxiety, depression or grief severity (Ghesquiere et al., 2013). None of the support types were associated with changes in anxiety severity (Ghesquiere et al., 2013).

Differences in the need for social support were found between western and eastern countries (Panagiotopoulos et al., 2013; Tiedt et al., 2016; Xu et al., 2017). In eastern countries, the participants often lived with their married children and perceived support from them (Jeon et al., 2013; Tiedt et al., 2016; Xu et al., 2017). However, relationships with children only had an impact on depressive symptoms for widowers, whereas cohabitation with children only had an impact on such symptoms for widows (Jeon et al., 2013). Attending support groups decreased significantly with age (Ghesquiere et al., 2013).

#### 4.2.2 Struggling with poor physical health

Six of the included studies described the informants as having multimorbidity or chronic diseases that caused them to struggle with poor physical health (Agrawal & Keshri, 2014; DiGiacomo et al., 2013; Jeon et al., 2013; Perkins et al., 2016; Tiedt et al., 2016; Zhou & Hearst, 2016) (Table 4). Three of the studies mainly focused on depression but also included information on chronic diseases (DiGiacomo et al., 2013; Jeon et al., 2013; Tiedt et al., 2016). Another three studies mainly focused on chronic diseases but included mental health measures (Agrawal & Keshri, 2014; Perkins et al., 2016; Zhou & Hearst, 2016). Two studies demonstrated that long‐term illness had a negative impact on the participants’ daily functioning (DiGiacomo et al., 2013; Tiedt et al., 2016) (Table 4).

Agrawal and Keshri (2014) revealed that the prevalence of communicable diseases was lower among older widows compared with older widowers. Perkins et al. (2016) found that a widower who had impaired cognitive ability and a mental disorder experienced poorer health. Furthermore, they indicated that both recent and long‐term widows were at risk of poorer health compared with married women. There was no evidence that widowhood acted as a protective factor for either gender.

Age differences were revealed in three studies (Agrawal & Keshri, 2014; Perkins et al., 2016; Zhou & Hearst, 2016). Widowers’ physical health was in decline and dropped more rapidly after the age of 70 (Zhou & Hearst, 2016). In addition, two studies revealed that the prevalence of non‐communicable diseases increased significantly with age and the same pattern was observed for other types of disease (Agrawal & Keshri, 2014; Perkins et al., 2016).

## 5 DISCUSSION

Twelve studies from different parts of the world are included in this systematic review. Two themes emerged from the thematic analysis (Table 3): emotional challenges related to experiences of bereavement, depression and anxiety based on the sub‐theme social support as the main strategy for coping with emotional pain and suffering. The second theme was: struggling with poor physical health.

The emotional challenges related to the experience of bereavement, depression and anxiety are illustrated by the descriptions of the emotional pain and suffering that constitute the state of widowhood. Between 10%–20% of widowed persons reported that the bereavement had an impact on their quality of life (QOL; Bonanno et al., 2005). Bereavement triggers intense emotions, such as sadness, loneliness, meaninglessness and hopelessness. These emotions influence the health of widows/widowers, leading to a lack of energy, activity and pleasure, culminating in social withdrawal and isolation (Gillies & Neimeyer, 2006). For many years, the emotions following bereavement have been described as universal, exposing a person to a higher risk of mental health problems (Holm, 2009). Recent research by Barrett (2017) revealed that emotions are socially constructed and related to an individual's experiences, context and social relationships. In the early 20th century, Freud suggested that grief might resemble depression, anxiety and posttraumatic stress (Gillies & Neimeyer, 2006). It has been argued that the responses to widowhood constitute grief disorder (Prigerson & Maciejewski, 2005) including intrusive thoughts, memories and images of the loss. Bereavement seems to shatter a widowed person's world, evoking a sense of meaninglessness, unworthiness and overwhelming distress (Gillies & Neimeyer, 2006). Widowed persons attempt to survive by numbing themselves emotionally, but unwanted thoughts and memories still intrude when they try to make sense of their loss. Rebuilding the self can resolve this existential dilemma (Gillies & Neimeyer, 2006). Bereavement and grief share characteristics such as distress and depression that affect health (Jacobsen, Zhank, Block, Maciejewski, & Prigerson, 2010). A century ago Freud stated that self‐esteem, self‐loathing and suicidality are associated with depression, but not grief (Freud, 1989; Prigerson, Vanderwerker, & Maciejewski, 2008). A previous study has found that narrative intervention can be used effectively for complicated grief disorder (Barbosa, Sám, & Rocha, 2013). Older widowed persons have derived benefit from narrating their stories and working through their loss and grief (Barbosa et al., 2013; Boelen, Keijser, van den Hout, & van den Bout, 2007). Grief has many symptoms in common with depression (Latham & Prigerson, 2004; Simon et al., 2005). Prolonged grief disorder is distinguished by the severity and duration of symptoms, as well as the marked distress and disability they evoke (Prigerson et al., 2008). Mental stress caused by loss and grief can be understood as sadness and loneliness, as well as the need to cope with stressors (Stahl, Arnold, Chen, Anderson, & Schulz, 2016; Stroebe & Schut, 2010). Coping with the new role of being a widow/widower can be overwhelming and cause frustration in daily life that can influence health and increase the risk of early death (Stahl et al., 2016; Stroebe & Schut, 2010). Poor mental health can lead to more psychotropic medication, psychiatric visits and all‐cause mortality (Möller, Björkenstam, Ljung, & Yngwe, 2011).

Social support is often described as the help provided by one's social network (Haber, Cohen, Lucas, & Baltes, 2007). Structural support is given by persons in the social network and related to the frequency of contact within the network. Functional support includes emotional and instrumental support as well as perceptions of and judgements about the support. There is a cultural difference between eastern and western countries, despite the fact that an individual's emotional and pain system is described as universal and developed from pain mechanisms that originated millions of years ago. Barrett (2017) views emotions as social constructions. The emotional pain system has been explained by Panksepp and Watt (2011) as social cohesion, which forges bonds between infants and caretakers, fortifies friendships and sexual relationships and promotes social solidarity among groups of living species. Arousal of this system can be related to social attachments, thus explaining how much one misses someone and why depression hurts so much (Panksepp & Watt, 2011). Explanation of bereavement and depression seems to be rooted in the attachment theory (Bowlby, 1980), which can facilitate understanding that bereavement involves emotions and that it is not essential to distinguish depression from prolonged grief disorder. Similar to this review study, Guiaux, Van Tilburg, and Van Groenou (2007) found an increase in emotional and instrumental support during the first 2 years of widowhood that decreased over time.

Filial piety is one of the guiding principles of elder care in Chinese culture, emphasizing respect, loyalty and support for older parents (Mjelde‐Mossey, Chi, & Lou, 2005). Even in Japan, obligations to support elderly parents seem to be mainly rooted in traditional Confucian ideals, where the eldest son has financial responsibility for his parents’ home and property, while his wife is expected to assume caretaking responsibilities (Therborn, 2004). However, developments in recent decades have led scholars to believe that filial piety may be less pervasive in contemporary Japan (Traphagan, 2003). Powers, Bisconti, and Bergeman (2014) revealed that structural and functional support remained stable across the first 2 years loss. However, emotional and social support seemed to decrease over time.

*Struggling with poor physical health *is supported by previous research. Studies identified increased mortality from causes such as heart diseases, cirrhosis of the liver, accidents and suicide (Stroebe, Schut, & Stroebe, 2007). As shown in this review, poor physical health in widowhood is most apparent in eastern countries (Moon, Glymour, Vable, Liu, & Subramanian, 2014; Shor et al., 2012). Previous research reported that poor health and/or loneliness in widowhood can increase morbidity and mortality (van den Berg, Lindeboom, & Portrait, 2011; Williams, 2005; Wittstein et al., 2005). Poor mental health can result in suicide (Möller et al., 2011). van den Berg et al. (2011) found strong effects of widowhood on mortality because of the increase in multimorbidity. Interventions soon after bereavement are vital for the length and QOL in widowhood. Mortality has been referred to as “the widowhood effect” and different mortality‐related causes have been reported (Möller et al., 2011; Stroebe et al., 2007). The widowhood effect seems to increase the need for physical and mental health care to reduce the risk of death both in eastern and western countries. From a stress perspective, it is argued that the combined effects of spousal death, cardiovascular disease and/or depression could make widowed older adults vulnerable to early death (Stahl et al., 2016). Research on gender revealed that older widows are more likely than older widowers to suffer from multimorbidity (Agrawal & Keshri, 2014). Widows are more prone to poor health and financial insecurity (Williams, Baker, Allman, & Roseman, 2006). This is supported by three of the included studies (Perkins et al., 2016; Xu et al., 2017; Zhou & Hearst, 2016), especially in the eastern part of the world. Poor health has been associated with age, education and socio‐economic conditions (Agrawal & Keshri, 2014).

### 5.1 Methodological discussion of the included studies

This systematic review has limitations due to the quality of the included studies (Table 2), the fact that many have only a small sample (Table 1) and lack information about methodological characteristics (Table 1). In addition, no studies from the Nordic countries or from Africa were found. One must also take into consideration that different cultures could have a severe impact on elderly widows and widowers. Psychological well‐being seems to be neglected in countries such as Pakistan, India, China and South Korea. These countries appear to focus more on the curative rather than the preventive aspects of health care (Khan, 2012). Such differences can be a limitation when reviewing studies from different parts of the world.

Another methodological limitation that could increase bias involves emotions and the fact that the roles of widowers and widows are dynamic and socially constructed (Barrett, 2017; DiGiacomo et al., 2013; Williams et al., 2006). Gender differences are more apparent in societies with rigid gender roles such as South Korea, India, China and even Japan, as outlined in four of the included studies (Jeon et al., 2013; Perkins et al., 2016; Xu et al., 2017; Zhou & Hearst, 2016). In eastern countries, the economic burden of widowhood is far more apparent than in western countries. Poor socio‐economic status can be related to increased use of healthcare services (Agrawal & Arokiasamy, 2009; Halleröd & Gustafsson, 2011). In high‐income countries, a better health status has been reported for widows than for widowers. Such findings seem to be inconsistent with studies from low‐income countries, where marriage has been described as a benefit for men. Thus, widowhood seems to be influenced to a great extent by culture and ethnicity (Kleinmann, 2012; Zhou & Hearst, 2016).

Traditional gender roles in these countries have placed widows in a subordinate position to widowers and although they persist in the older South Korean and Chinese population, they seem to be weakening (Jeon et al., 2013). Culture, gender and age appear to play an important role in the experience of loss (Jeon et al., 2013; Xu et al., 2017; Zhou & Hearst, 2016). Stereotypes of older people need to be considered because of the differences in what is acceptable behaviour for widows and widowers in western and eastern countries. An important point to bear in mind is that such differences make it difficult to draw conclusions about the health burden in countries not included in this review.

## 6 CONCLUSION

Healthcare professionals need more knowledge and skills to address the factors that influence the health of older widowed adults. Future research should explore the lived experiences of the health problems and burden of widowhood. Healthcare professionals must take action to minimize the risk of mortality due to health problems and multimorbidity in this population. It is important to build a community team that can contact widows and widowers in the first year of bereavement to map their unmet need for help, thus preventing prolonged grief, physical health problems and mortality.

## 7 CONTRIBUTIONS

ALH: Study design, ALH: data collection, and ALH, AB and ES: data analysis, discussion and preparation.

## ETHICAL APPROVAL

As this is a systematic review of previous studies, a patient consent statement and Research Ethics Committee approval were not required.

## CONFLICT OF INTEREST

The authors state that there is no conflict of interest to declare.
